# Supercapacitor Separators from Upcycled Waste Paper with Functionalized Surfaces

**DOI:** 10.3390/mi17030310

**Published:** 2026-02-28

**Authors:** Min Jun Lee, Inho Cho, Kwang Se Lee

**Affiliations:** 1 Department of Materials Science and Engineering, Gyeongkuk National University, Andong 36729, Republic of Korea; dlalswns1927@gmail.com; 2 Secondary Battery Convergence and Open Sharing System, Kyungnam College of Information & Technology, Busan 47011, Republic of Korea; inhocho92@eagle.kit.ac.kr

**Keywords:** supercapacitors, separator, waste newspaper, polyaniline (PANI) coating

## Abstract

This study presents a sustainable strategy for developing high-performance supercapacitor separators through the upcycling of waste newspapers into functional cellulose-based membranes. The intrinsic porous architecture of cellulose fibers was exploited as a robust scaffold, onto which Parylene C and polyaniline (PANI) layers were sequentially introduced to reinforce mechanical integrity and enhance electrochemical functionality. The resulting dual-layer configuration exhibited significantly improved interfacial stability and ion-transport characteristics compared with conventional polyethylene separators. Comprehensive structural and electrochemical analyses verified that the synergistic combination of Parylene C and PANI coatings effectively optimized separator–electrolyte interfacial properties and reduced impedance. Beyond performance enhancement, this work establishes an environmentally responsible route for valorizing paper waste, offering a viable pathway toward sustainable energy storage technologies.

## 1. Introduction

Growing global energy consumption and the associated environmental burden have intensified the need for sustainable energy production and storage technologies [1,2,3]. Among various energy storage options, supercapacitors (SCs) are particularly attractive because they offer high power density, rapid charge–discharge capability, and long cycle life, making them suifigure for applications that require fast energy delivery and extended durability [4]. In these devices, separators play a decisive role by providing an ion-transport pathway while electrically isolating the electrodes, thereby ensuring both performance and safety [5].

Recent studies have also emphasized safe and stable operation of supercapacitors through materials and electrolyte design [6]. An ideal separator must exhibit mechanical robustness, dimensional and thermal stability, appropriate porosity, and reliable electrolyte wettability, while maintaining strict electronic insulation [7,8]. Commercial SC separators are typically porous polyolefin membranes (e.g., Polyethylene (PE)/Polypropylene (PP)) or cellulose-based papers, whereas ion-exchange membranes such as Nafion are more widely used as proton-conducting membranes in other electrochemical systems and have been explored only in specific SC configurations [9,10]. Although Nafion provides excellent ionic conductivity and chemical stability, its fossil-based origin and high cost restrict its suitability for large-scale, low-carbon deployment [11,12,13]. These limitations have motivated the development of natural or bio-derived membrane materials as more sustainable separators for supercapacitor systems.

Cellulose has emerged as a promising separator material owing to its natural abundance, porosity, and favorable thermal and chemical stability [14]. In particular, waste newspaper represents an attractive cellulose source because its fibrous structure can be re-engineered into porous membranes, enabling the upcycling of low-value paper waste into high-value functional components for energy storage devices [15,16,17]. Nevertheless, cellulose-based separators can be susceptible to mechanical damage, and their electrochemical performance is highly sensitive to surface chemistry and pore structure. These limitations highlight the need for rational surface engineering strategies that can simultaneously stabilize the cellulose framework and tailor ion-transport properties.

In previous work, cellulose separators prepared from recycled newspaper were coated with a vapor-deposited polymer layer to improve their mechanical integrity and interfacial stability [18]. The conformal coating effectively reinforced the fibrous network and increased surface hydrophobicity, demonstrating that polymeric surface modification is a viable route to enhance the durability and safety of cellulose-based separators. Parylene C, in particular, is a polymer with excellent physicochemical properties and microfabrication capabilities that can be deposited as a conformal coating onto a wide range of substrates, and has been widely employed in electronics, semiconductor devices, and biomedical systems [19,20]. Its high hydrophobicity has also been exploited in biomedical applications, such as biosensor surface modifications designed to improve stability and control biomolecular interactions [21,22]. However, although this mechanically robust architecture improves structural stability and interfacial protection, it still provides limited opportunities to actively regulate ionic conductivity, interfacial resistance, and ion-transport behavior, which are crucial for achieving high-performance supercapacitors.

Previous studies have incorporated functional polymers such as polypyrrole, poly(3,4-ethylenedioxythiophene), and polyaniline into supercapacitor electrodes and related components to increase the number of ion-accessible sites and improve electrolyte wettability under mild processing conditions [23,24,25,26]. These reports highlight that such polymers are effective in tailoring interfacial properties within energy-storage devices. However, their use as surface-modifying layers on porous separators has rarely been explored. In particular, polyaniline (PANI) has been widely studied as a versatile functional polymer because it can form relatively thin and uniform layers on various substrates, exhibits good chemical and environmental stability, and can be synthesized through relatively simple solution-based routes [27,28,29]. When employed as a surface-modifying layer on porous membranes, PANI can enhance electrolyte affinity and facilitate more efficient ion-transport pathways at the electrolyte–separator interface, thereby improving the interfacial environment for ion migration in supercapacitor systems.

Building on the mechanically reinforced cellulose separator established in the earlier study, this work proposes a multilayer separator architecture that upcycles waste newspaper into an environmentally friendly, high-performance separator for supercapacitors. A Parylene C coating is first applied to the recycled-cellulose scaffold to provide mechanical robustness, dimensional stability, and electronic insulation, and a PANI-based functional polymer layer is subsequently introduced on top of this polymer-coated framework. In this design, the inner cellulose/Parylene C framework maintains the required mechanical strength and electronic insulation, while the outer PANI layer is utilized to improve electrolyte interaction and ionic transport at the separator–electrolyte interface. The objective of this study is to demonstrate that integrating PANI into a recycled-cellulose-based separator enables simultaneous enhancement of the mechanical stability and ion-transport properties of the separator, thereby providing a sustainable and functionally advanced separator platform for high-performance supercapacitors.

## 2. Materials and Methods

### 2.1. Preparation of Newspaper Separator (NP) and Parylene-C-Coated Newspaper (PC@NP)

Waste newspaper (The Korea Herald) was selected as the cellulose-based substrate for separator fabrication owing to its porous fiber network and good electrolyte permeability. The printed sheets were cut into pieces with dimensions suitable for use as separator substrates, and the as-cut samples are denoted as NP (newspaper). Prior to coating, NP substrates were gently dried to remove residual moisture. Parylene C was deposited onto NP by chemical vapor deposition (CVD) using a commercial CVD system (PDS 2010, Specialty Coating Systems, Indianapolis, IN, USA) with di-para-xylylene dimer (Parylene C dimer, Specialty Coating Systems, USA, >98% purity) as the precursor. During deposition, the precursor zone, pyrolysis furnace, and deposition chamber were maintained at approximately 175 °C, ~690 °C, and room temperature (~25 °C), respectively. After evacuation to high vacuum (~10^−2^ Pa), the system pressure was adjusted to ~15 Pa so that the reactive Parylene C species could polymerize on the newspaper surface. Under these conditions, a conformal Parylene C layer was formed throughout the porous cellulose network, yielding a mechanically reinforced Parylene-C-coated newspaper separator, denoted as PC@NP.

### 2.2. Preparation of PANI-Coated Parylene-C Newspaper (PANI@PC@NP)

**Figure 1:**
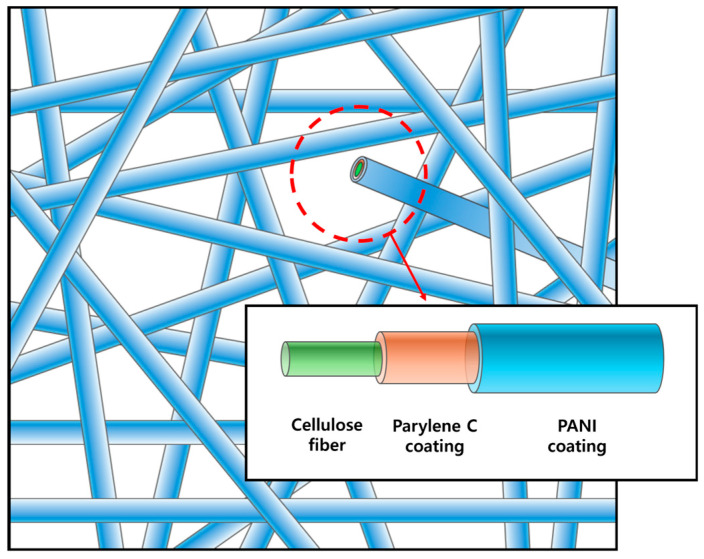
Schematic illustration of the multilayer-coated cellulose separator (PANI@PC@NP) architecture.

To tailor the interfacial ion transport and electrochemical response of the separator, a polyaniline (PANI) layer was formed on PC@NP by in situ chemical polymerization. Aniline monomer (99.5%, Sigma-Aldrich, St. Louis, MO, USA) was dissolved in an aqueous perchloric acid solution (HClO_4_, ACS reagent, 70%, Sigma-Aldrich; 0.50 M) to prepare the monomer solution (0.10 M aniline), and an oxidant solution containing ammonium persulfate (APS, reagent grade, 98%, Sigma-Aldrich) was prepared separately in the same acid medium (0.50 M HClO_4_, 0.11 M APS), corresponding to an aniline: APS molar ratio of 1:1.10. PC@NP substrates were immersed in the monomer solution, after which the oxidant solution was added under stirring. The polymerization was carried out at 0 °C for 4 h under controlled conditions to promote the formation of a thin PANI layer on the Parylene C surface and within the porous network. After polymerization, the PANI-modified separators were thoroughly rinsed with deionized water until the washings became colorless, in order to remove residual monomer and by-products, and then dried at room temperature prior to further characterization. The resulting PANI-coated Parylene-C newspaper separator is denoted as PANI@PC@NP (PANI-coated Parylene-C newspaper). The multilayer architecture of the cellulose fiber, consisting of a cellulose core, an intermediate Parylene C coating, and an outer PANI layer, is schematically illustrated in Figure 1.

### 2.3. Physical and Chemical Characterization

The surface morphology and coating uniformity of PE (Celgard), PC@NP, and PANI@PC@NP were examined by field-emission scanning electron microscopy (SEM, SNE-4500 M Plus, SEC, Korea). Nanoscale surface topography and roughness were further analyzed using atomic force microscopy (AFM, NX10, Park Systems, Korea) operated in non-contact mode. The chemical structure and bonding states of the coated separators were investigated using Fourier-transform infrared spectroscopy (FTIR, Cary 630, Agilent Technologies, USA). FTIR spectra were recorded in the wavenumber range of 4000–500 cm^−1^ to identify characteristic absorption bands corresponding to Parylene C and PANI on the cellulose substrate. Surface wettability was evaluated via static water contact angle measurements with deionized water using a contact angle goniometer (DSA25, KRÜSS, Germany) at room temperature. A 3 µL droplet of deionized water was dispensed onto the separator surface, and the contact angle was recorded after 10 s. The reported contact angle values were obtained by averaging measurements acquired at multiple positions on each sample. Thermal dimensional stability was assessed by a thermal shrinkage test; square specimens were heated at 150 °C for 15 min, and the side length was measured before $L_{0}$ and after L heat treatment. The linear shrinkage ratio was calculated as

(1) $$Shrinkage\;ratio(S)=(L_{0}-L)/L_{0}\times 100$$

where $L_{0}$ and L denote the initial and final side lengths, respectively.

### 2.4. Electrochemical Measurements

The electrochemical response of PE (Celgard), PC@NP, and PANI@PC@NP was evaluated to compare separator–electrolyte interfacial behavior using a potentiostat/galvanostat. All measurements were conducted in an aqueous 1 M H_2_SO_4_ electrolyte using a three-electrode configuration. A separator disc (12 mm in diameter) was placed on a glassy carbon substrate to provide stable current-collecting support for probing the separator–electrolyte interface in a three-electrode setup; Ag/AgCl and Pt wire were used as the reference and counter electrodes, respectively. The exposed geometric area of the working electrode was fixed using an insulating O-ring/mask, and the coated side of the separator (when applicable) faced the electrolyte. Prior to testing, the separator discs were fully wetted in the electrolyte for 12 h to ensure stable interfacial contact. Cyclic voltammetry (CV) was performed in the potential window of 0.0–1.2 V (vs. Ag/AgCl) at scan rates of 30–100 mV s^−1^. Galvanostatic charge–discharge (GCD) tests were conducted within the same potential window at constant currents of 1.0, 1.5, and 2.0 mA. The specific capacitance was calculated from the discharge branch according to

(2) C=(I×Δt)/(m×ΔV)

where I is the applied current, Δt is the discharge time, m is the mass of the separator disc used for normalization in the present geometry, and ΔV is the effective potential change excluding the initial IR drop. Electrochemical impedance spectroscopy (EIS) was carried out at the open-circuit potential with an AC amplitude of 10 mV over a frequency range of 100 kHz to 0.1 Hz. EIS data were compared using Nyquist-plot descriptors, including the high-frequency intercept and overall impedance features, for consistent evaluation among samples. The potential window was selected based on stable CV and GCD profiles without noticeable parasitic reactions during testing.

## 3. Results

**Figure 2:**
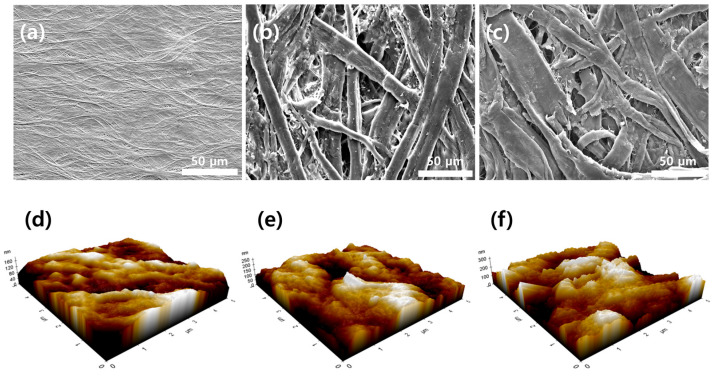
SEM and AFM characterization of coating-induced surface evolution. (**a**–**c**) SEM micrographs and (**d**–**f**) AFM 3D topography images of PE, PC@NP, and PANI@PC@NP. In the AFM images, the color scale indicates relative height variation.

The morphologies of the separators were examined using SEM. As shown in Figure 2a, the PE separator exhibits a smooth and dense morphology, which is characteristic of commercial polyolefin separators and is used here as a baseline reference. After applying a conformal Parylene C coating on the cellulose-based substrate (PC@NP, Figure 2b), the fibrous framework becomes uniformly encapsulated and the apparent fiber thickness increases, consistent with conformal deposition of the polymer layer. Notably, Figure 2b,c correspond to the cellulose-based substrate after sequential coating (PC@NP and PANI@PC@NP), whereas Figure 2a shows the commercial PE reference. This coating partially fills inter-fiber voids and renders the inter-fiber structure more compact, which is expected to improve structural robustness and electrolyte retention. With the additional PANI coating (PANI@PC@NP, Figure 2c), the surface becomes more aggregated and interconnected, forming a denser network. Overall, the progressive evolution from PE to the sequentially coated configurations demonstrates that stepwise surface engineering substantially alters the microstructure and inter-fiber architecture, suggesting improved electrochemical suitability for energy storage applications. Based on the SEM observations, the Parylene C coating appears to stabilize the cellulose network and modify the separator surface/interface, while the additional PANI layer forms a secondary interfacial coating. These observations support the interpretation of the electrochemical results in the separator-mounted configuration.

As shown in the AFM image (Figure 2d), the PE separator exhibited a relatively smooth surface, with a root mean square (RMS) roughness of 27.171 nm (Table 1). This smooth morphology, characteristic of commercial polyolefin membranes, contributes to mechanical robustness but provides limited surface heterogeneity and wettability, which may restrict electrolyte uptake and interfacial contact with the electrode. After Parylene C coating on the cellulose-based separator (PC@NP) (Figure 2e), the surface roughness increased significantly, with an RMS roughness of 41.426 nm. This enhancement is attributed to the uniform deposition of the Parylene C polymer layer, which effectively encapsulated the cellulose fibers, reinforcing the mechanical integrity of the separator. The increased roughness also indicates modified surface topography, which can promote electrolyte retention and interfacial stability. Following the additional PANI coating (PANI@PC@NP) (Figure 2f), the surface roughness further increased, reaching an RMS roughness of 55.320 nm. The AFM image shows that the PANI layer formed a conformal coating with well-distributed surface features, increasing the effective surface area. This morphological change can improve electrolyte affinity and interfacial contact, facilitating ion transport across the separator interface. Overall, the progressive increase in surface roughness from PE to the sequentially coated configurations demonstrates the systematic influence of surface modification on surface topography and interfacial properties.

**Figure 3:**
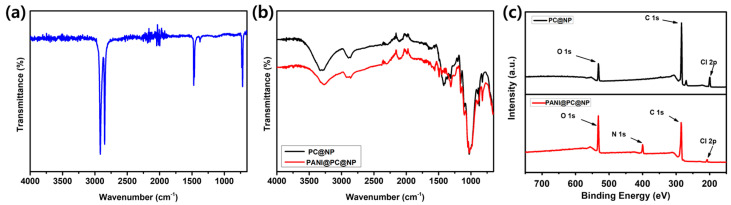
FTIR spectra of (**a**) PE and (**b**) PC@NP and PANI@PC@NP, and (**c**) XPS survey spectra of PC@NP and PANI@PC@NP.

To investigate the chemical composition and surface functionalization of the separators, FTIR analysis was conducted, and the spectra are presented in Figure 3. The spectra reflect progressive chemical changes associated with sequential Parylene C and PANI coating. As shown in Figure 3a, the PE separator exhibits characteristic bands of a non-polar hydrocarbon backbone, including strong C–H stretching absorptions in the 2800–3000 cm^−1^ region, while showing no prominent signals attributable to polar functional groups, consistent with the chemically inert nature of polyethylene. Accordingly, PE is used as a practical baseline for evaluating coating-induced spectral changes. After coating the cellulose-based substrate with Parylene C (PC@NP), additional FTIR absorptions appear in the ~800–900 cm^−1^ region (assignable to C–H bending vibrations) and near ~1200 cm^−1^ (attributable to C–Cl stretching), together with C–H stretching bands around ~2850 and ~2920 cm^−1^, supporting the presence of the Parylene C layer (Figure 3b). Consistently, XPS spectra of PC@NP (Figure 3c) show a clear Cl 2p signal along with the dominant C 1s and O 1s peaks, indicating the introduction of the chlorine-containing Parylene C coating. The broad band near ~3300 cm^−1^, mainly associated with O–H stretching in the cellulose framework, becomes less intense after coating, which can be interpreted as partial coverage of surface hydroxyl groups. With the subsequent PANI coating (PANI@PC@NP), further spectral changes are observed in the 1450–1600 cm^−1^ region, which can be assigned to ring-related vibrations of the PANI framework (benzenoid/quinoid structures), along with a broad band in the ~3200–3400 cm^−1^ region that can include N–H stretching contributions (Figure 3b). This region is generally dominated by hydrogen-bonded stretching vibrations, and thus the broad envelope can reflect overlapping contributions from the cellulose O–H framework and the N–H moieties introduced by the PANI overlayer. To complement the FTIR interpretation, an additional N 1s signal appears in the XPS spectrum of PANI@PC@NP (Figure 3c), indicating the presence of nitrogen-containing surface functionalities introduced by PANI. In the XPS survey comparison after PANI coating, the Cl 2p signal from the underlying Parylene C layer is attenuated and the O 1s signal shows a noticeable change, which is consistent with overlayer coverage on the surface. Together, the FTIR and XPS results capture the progressive surface-chemistry changes induced by the sequential coating process.

**Figure 4:**
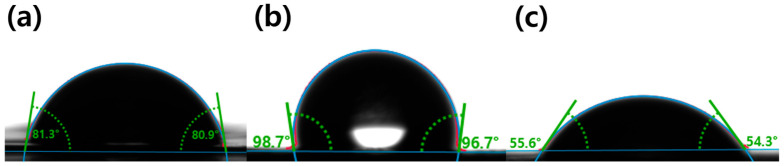
Water contact angle measurements of the separators. (**a**) PE, (**b**) PC@NP, and (**c**) PANI@PC@NP. The colored overlays indicate the fitted droplet profile and the baseline used for contact-angle determination.

The wettability of the separators was evaluated by contact angle measurements to examine changes in surface characteristics that can influence liquid interaction at the separator interface. Compared with PE (Figure 4a), the separator surface was clearly altered after Parylene C coating (Figure 4b), showing an increased contact angle and thus a reduced hydrophilicity. This trend suggests that the uniformly deposited Parylene C layer acts as a hydrophobic protective barrier while reinforcing the cellulose network, which is consistent with improved structural robustness of the coated separator. After the additional PANI coating (Figure 4c), the contact angle decreased to ~55.98° (Table 2), indicating an enhanced surface wettability relative to PC@NP and implying a more favorable interfacial contact with the electrolyte. Overall, the sequential coating process enabled a stepwise transition from an uncoated porous surface to a structurally reinforced, more hydrophobic interface, followed by the formation of a more wettable surface after PANI deposition. The final PANI@PC@NP therefore achieves a balanced combination of structural stability and interfacial wettability, supporting its potential as a functional separator for high-performance energy storage applications.

**Figure 5:**
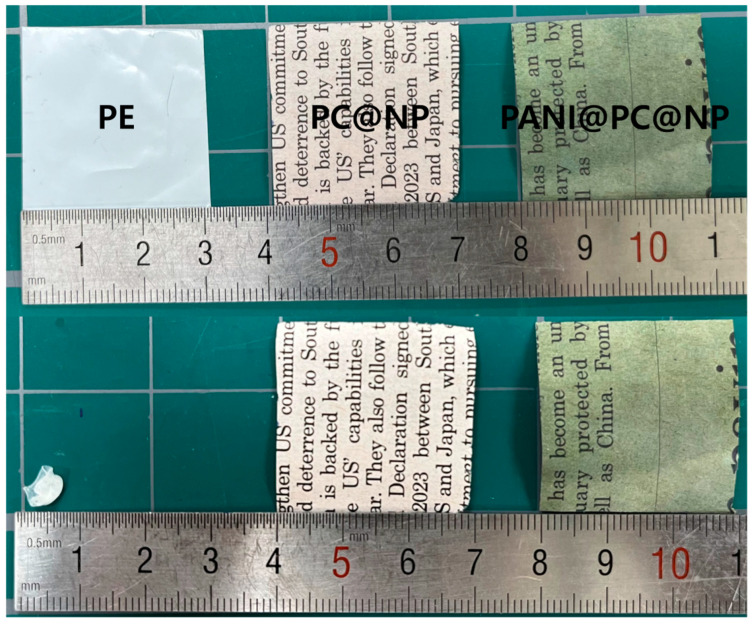
Photographs of PE, PC@NP, and PANI@PC@NP separators before (**top**) and after (**bottom**) heat treatment showing their thermal shrinkage behavior.

As illustrated in Figure 5, all separators exhibit comparable in-plane dimensions before heating, with an initial side length of approximately 3.0 cm. After the thermal treatment, the PE separator undergoes severe dimensional collapse, and its side length decreases to about 0.6 cm, corresponding to a linear shrinkage ratio of ~80%. In contrast, both PC@NP and PANI@PC@NP maintain their macroscopic dimensions within the resolution of optical inspection, and no measurable shrinkage is detected (S ≈ 0%). These results indicate that the cellulose-based framework, reinforced by the conformal Parylene C coating and further modified with the PANI surface layer, effectively suppresses heat-induced shrinkage and ensures excellent macroscopic dimensional stability. Importantly, the introduction of PANI does not compromise the inherent thermal robustness of the polymer-coated cellulose separator, which is a critical requirement for reliable operation of supercapacitor devices under elevated temperature conditions.

**Figure 6:**
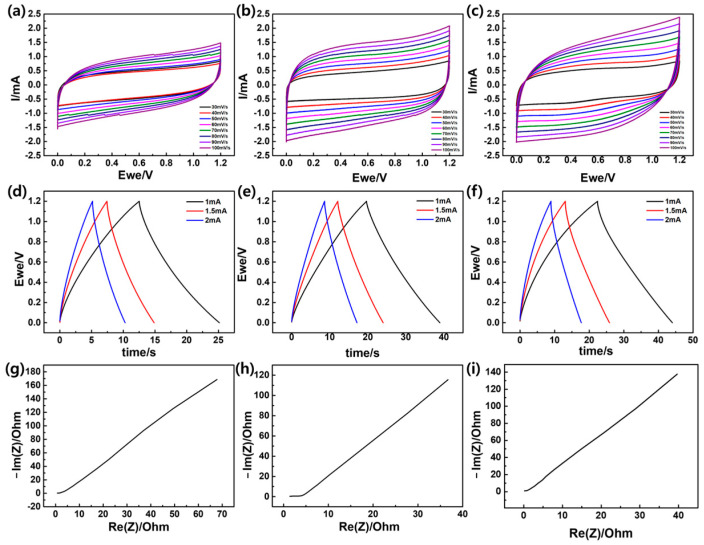
Electrochemical performance of PE, PC@NP, and PANI@PC@NP separators evaluated by cyclic voltammetry (CV), galvanostatic charge–discharge (GCD), and electrochemical impedance spectroscopy (EIS). (**a**–**c**) CV curves at various scan rates, (**d**–**f**) GCD profiles at different current densities, and (**g**–**i**) Nyquist plots.

In this separator-mounted configuration, the electrochemical trends are interpreted primarily in terms of separator/interface effects. The PANI overlayer is therefore discussed as an interfacial modifier that improves wetting/contact and ion transport, rather than as an active component providing a dominant pseudocapacitive contribution to the device response. Accordingly, the electrochemical discussion focuses on comparative separator/interface trends under a consistent testing protocol, and a detailed deconvolution of possible PANI-specific redox features is therefore beyond the scope of the present discussion. Specifically, the Parylene C layer mainly provides structural/interfacial stabilization of the cellulose framework, whereas the additional PANI layer mainly improves interfacial contact and ion-transport behavior by tuning the separator surface. Cyclic voltammetry (CV) was carried out to probe the electrochemical response of the separators in terms of electrolyte accessibility and ion-transport behavior. Figure 6 compares the CV curves of (a) PE, (b) PC@NP, and (c) PANI@PC@NP, showing a systematic increase in current response with sequential surface modification. The PE separator (Figure 6a) exhibits a nearly rectangular profile within the selected potential window, which is typically associated with capacitive behavior. However, the overall current response is relatively low, suggesting limited ionic accessibility and transport at the separator–electrolyte interface. After conformal deposition of Parylene C on the cellulose-based substrate (PC@NP, Figure 6b), the current response increases markedly, reflecting enhanced interfacial stability and a more continuous electrolyte-filled percolation network for ion transport sustained by the mechanically reinforced porous scaffold. The CV curves maintain a quasi-rectangular, capacitive-like shape, consistent with improved electrolyte accessibility and reduced polarization at the separator–electrolyte interface. The PANI@PC@NP separator (Figure 6c) shows the highest current response among the samples while preserving the overall capacitive profile. This enhancement can be attributed to the PANI overlayer, which promotes electrolyte affinity and facilitates ion transport across the separator–electrolyte interface, thereby mitigating polarization and resistive losses. Overall, the CV results demonstrate that stepwise surface modification progressively optimizes separator-related interfacial and transport properties, leading to a more favorable electrochemical response during operation.

Galvanostatic charge–discharge (GCD) measurements were conducted in the same potential window to compare constant-current polarization and discharge responses associated with separator–electrolyte interfacial transport. Figure 6 presents the GCD profiles of (d) PE, (e) PC@NP, and (f) PANI@PC@NP, revealing progressively reduced polarization and improved ion-transport characteristics as the separator surface is sequentially modified. The PE separator (Figure 6d) exhibited a rapid voltage drop and a short discharge time, corresponding to a lower specific capacitance (5.436 F g^−1^) in the separator-mounted configuration (Table 3). Without additional surface modification, PE shows limited performance due to restricted electrolyte accessibility at the separator–electrolyte interface and higher interfacial polarization, which hampers effective ion transport during current passage. This limitation is consistent with the well-known drawbacks of polyolefin separators: the nonpolar and highly crystalline PE matrix typically exhibits limited electrolyte affinity/uptake and a less ion-accessible porous network, which increases interfacial/ohmic resistance in aqueous electrolytes and manifests as a larger iR drop and stronger polarization during GCD. Following Parylene C coating on the cellulose-based separator (PC@NP, Figure 6e), a substantial increase in capacitance was observed, with a recorded specific capacitance (8.272 F g^−1^), consistent with improved structural integrity and stabilized ion-transport pathways. The extended discharge time suggests that the coating improves ionic diffusion and interfacial ion transport, thereby reducing polarization during charge–discharge. The Parylene C layer provided a more stable and uniform interface, reducing internal resistance and enabling smoother ion migration. Overall, the GCD profiles are largely characteristic of non-faradaic polarization, indicating that the observed differences primarily originate from improved interfacial ion transport and reduced polarization losses. The most pronounced enhancement was observed for PANI@PC@NP, showing the longest discharge duration and the highest specific capacitance (9.358 F g^−1^) under the same separator-mounted geometry, consistent with reduced polarization and improved electrolyte accessibility. The change in the GCD response indicates that the PANI layer improves the interfacial environment for ion migration, which is consistent with enhanced ion accessibility and reduced polarization during charge–discharge. The incorporation of PANI improves charge retention and enhances ion accessibility by promoting electrolyte uptake and facilitating ion transport across the separator–electrolyte interface. As a result, PANI@PC@NP exhibits improved stability upon repeated GCD cycling, as evidenced by higher capacitance retention, consistent with a more favorable separator–electrolyte interfacial environment. Overall, these results indicate that sequential surface modification systematically improves separator-related interfacial properties and ion-transport behavior, which translates into more stable cell operation. While Parylene C improves electrochemical behavior by optimizing surface morphology and ion diffusion, the introduction of PANI further enhances the electrochemical response by improving electrolyte affinity and interfacial ion transport. The combined improvement in ion transport and interfacial resistance in PANI@PC@NP yields the most effective electrochemical response among the tested separators, supporting its potential as a functional separator for supercapacitor operation.

Electrochemical impedance spectroscopy (EIS) was employed to evaluate the impedance characteristics associated with ionic transport and interfacial processes of the separators. Figure 6g–i shows the Nyquist plots of PE, PC@NP, and PANI@PC@NP, indicating an overall reduction in impedance after sequential surface modification. As the spectra do not exhibit a well-resolved semicircle, the impedance characteristics were compared using the real-part values at identical imaginary components. At −Im(Z) = 50 Ω, Re(Z) decreases from ~25.1 Ω for PE to ~22.5 Ω for PC@NP and further to ~17.1 Ω for PANI@PC@NP, and a consistent trend is observed at −Im(Z) = 100 Ω (~41.2, ~35.6, and ~29.3 Ω, respectively). In addition, the low-frequency response becomes progressively more vertical with coating, supporting reduced resistive losses and more continuous ion-transport pathways in the modified separators. Overall, these results substantiate that the stepwise coating strategy mitigates impedance contributions in the separator–electrolyte system and improves the electrochemical suitability of the cellulose-based separator for supercapacitor operation.

**Figure 7:**
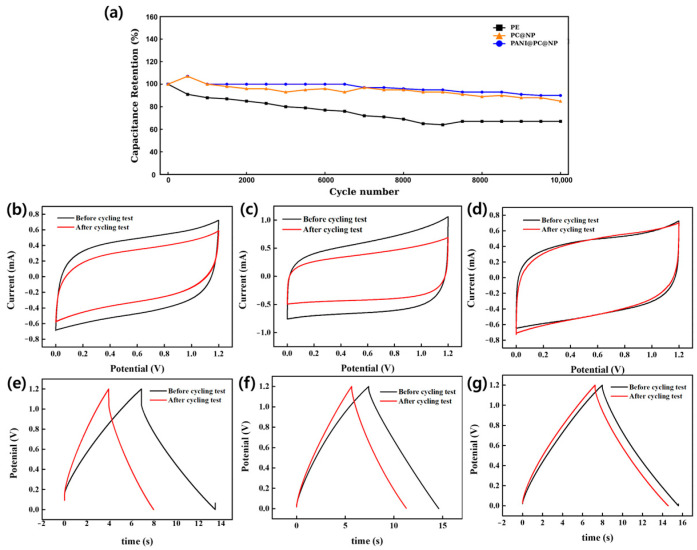
Long-term cycling stability and electrochemical with PE, PC@NP, and PANI@PC@NP. (**a**) Capac-itance retention as a function of cycle number up to 10 000 cycles. (**b**–**d**) Cyclic voltammograms recorded before and after cycling for (**b**) PE, (**c**) PC@NP, and (**d**) PANI@PC@NP separa-tor-mounted electrodes. (**e**–**g**) Galvanostatic charge–discharge profiles measured before and after cycling for (**e**) PE, (**f**) PC@NP, and (**g**) PANI@PC@NP separator-mounted electrodes.

The long-term cycling durability of the PE, PC@NP, and PANI@PC@NP separators was evaluated by repeated galvanostatic charge–discharge cycling up to 10,000 cycles, and the capacitance retention is summarized in Figure 7a. While all samples show comparable initial behavior, the PE separator exhibits a continuous decline in retention, reaching ~70% after 10,000 cycles. In contrast, the coated separators (PC@NP and PANI@PC@NP) maintain substantially higher retention (approximately >85–90%) over the same cycling window, indicating improved stability under prolonged cycling. The CV curves recorded before and after the cycling test (Figure 7b–d) further reflect the durability of each separator. After cycling, PE shows a more pronounced change in the CV response, whereas the coated separators largely preserve the overall CV shape with comparatively smaller variations, supporting their improved electrochemical stability during prolonged operation. Consistent trends are also observed in the GCD profiles before and after cycling (Figure 7e–g). PE exhibits more evident changes in the charge–discharge response after cycling, whereas PC@NP and PANI@PC@NP show relatively minor deviations, indicating better retention of electrochemical behavior. Overall, the cycling results (Figure 7a–g) demonstrate that sequential surface modification improves durability under repetitive cycling, with PANI@PC@NP showing the most stable response among the tested separators. Overall, the cycling results demonstrate that sequential surface modification effectively enhances the durability of the cellulose-based separators under prolonged operation. The coated separators exhibit smaller deviations in both CV and GCD responses after cycling, consistent with improved electrochemical stability. Among the tested samples, PANI@PC@NP shows the most stable behavior across the cycling evaluation.

## 4. Conclusions

In this study, a cellulose-based separator derived from waste newspaper was sequentially coated with Parylene C and polyaniline (PANI), and its morphological, interfacial, and electrochemical characteristics were evaluated in comparison with a commercial PE separator. The results indicate a stepwise improvement in performance with increasing surface modification. AFM analysis confirmed that the coated separators exhibited progressively increased surface roughness from PE to PC@NP and further to PANI@PC@NP, reflecting the formation of conformal polymeric layers. Contact angle measurements verified that surface wettability was effectively tuned through coating, supporting improved electrolyte interaction and interfacial stability. Electrochemical analyses (CV, GCD, and EIS) consistently demonstrated reduced impedance and improved interfacial ion-transport characteristics for the coated separators compared with PE, with PANI@PC@NP showing the most pronounced improvement. These findings suggest that sequential surface modification of the cellulose-based separator is an effective strategy to enhance separator functionality and offers a sustainable route toward high-performance separator materials for energy storage applications.

## Figures and Tables

**Table 1:** AFM-derived surface roughness (RMS) of PE, PC@NP, and PANI@PC@NP corresponding to Figure 2d–f.

Separator	RMS (nm)
PE	27.171
PC@NP	41.426
PANI@PC@NP	55.320

**Table 2:** Water contact angle values (mean ± SD) of PE, PC@NP, and PANI@PC@NP corresponding to Figure 4.

Separator	Contact Angle (°)
PE	80.8 ± 0.59
PC@NP	97.51 ± 0.789
PANI@PC@NP	55.98 ± 0.543

**Table 3:** Specific capacitance at different applied currents.

Applied Current (mA)	PE (F/g)	PC@NP (F/g)	PANI@PC@NP (F/g)
1.0	5.436	8.272	9.358
1.5	4.814	7.706	8.283
2.0	4.451	7.316	7.604

## Data Availability

The data presented in this study are available on request from the corresponding author. The data are not publicly available due to institutional policy and ongoing related research.

## Abbreviations

The following abbreviations are used in this manuscript:

SCs	Supercapacitors
PE	Polyethylene
NP	Newspaper
PC	Parylene C
PANI	Polyaniline
PC@NP	Parylene C coated newspaper separator
PANI@PC@NP	PANI coated Parylene C newspaper separator
CVD	Chemical vapor deposition
APS	Ammonium persulfate
SEM	Scanning electron microscopy
AFM	Atomic force microscopy
FTIR	Fourier-transform infrared spectroscopy
CV	Cyclic voltammetry
GCD	Galvanostatic charge–discharge
EIS	Electrochemical impedance spectroscopy
RMS	Root mean square
SD	Standard deviation

## References

[1] Manthiram A. (2020). A reflection on lithium-ion battery cathode chemistry. Nat. Commun..

[2] Liu J., Hull V., Godfray H.C.J., Tilman D., Gleick P., Hoff H., Pahl-Wostl C., Xu Z., Chung M.G., Sun J. (2018). Nexus approaches to global sustainable development. Nat. Sustain..

[3] Comello S., Reichelstein S. (2019). The emergence of cost effective battery storage. Nat. Commun..

[4] Ahankari S., Lasrado D., Subramaniam R. (2022). Advances in materials and fabrication of separators in supercapacitors. Mater. Adv..

[5] Wang H., Zhou Q., Yao B., Ma H., Zhang M., Li C., Shi G. (2018). Suppressing the Self-Discharge of Supercapacitors by Modifying Separators with an Ionic Polyelectrolyte. Adv. Mater. Interfaces.

[6] Li S., Tian Q., Chen J., Chen Y., Guo P., Wei C., Cui P., Jiang J., Li X., Xu Q. (2023). An intrinsically non-flammable organic electrolyte for wide temperature range supercapacitors. Chem. Eng. J..

[7] Li J., Jia H., Ma S., Xie L., Wei X.X., Dai L., Wang H., Su F., Chen C.J. (2022). Separator Design for High-Performance Supercapacitors: Requirements, Challenges, Strategies, and Prospects. ACS Energy Lett..

[8] Wang X., Zheng W., Zhao H., Li J., Chen S., Xu F. (2025). Robust and High-Wettability Cellulose Separators with Molecule-Reassembled Nano-Cracked Structures for High-Performance Supercapacitors. Nano-Micro Lett..

[9] Agarwal T., Prasad A.K., Advani S.G., Babu S.K., Borup R.L. (2024). Infrared spectroscopy for understanding the structure of Nafion and its associated properties. J. Mater. Chem. A.

[10] Loise V., Simari C. (2025). Next-Generation Nafion Membranes: Synergistic Enhancement of Electrochemical Performance and Thermomechanical Stability with Sulfonated Siliceous Layered Material (sSLM). Polymers.

[11] Komers F., Plachá D., Bruggen B.V., Velizarov S. (2025). Towards Sustainable Proton Exchange Membranes: Materials and Challenges for Water Electrolysis. Polymers.

[12] Maiti T.K., Singh J., Dixit P., Majhi J., Bhushan S., Bandyopadhyay A., Chattopadyay S. (2022). Advances in perfluorosulfonic acid-based proton exchange membranes for fuel cell applications: A review. Chem. Eng. J. Adv..

[13] Shawalludin N.S.F., Sha’rani S.S., Suhot M.A., Sarip S., Nasef M.M. (2025). Cellulose-Based Ion Exchange Membranes for Electrochemical Energy Systems: A Review. Membranes.

[14] Das M., Das P.S., Pramanik N.C., Basu R.N., Raja M.W. (2023). Advanced Sustainable Trilayer Cellulosic “Paper Separator” Functionalized with Nano-BaTiO_3_ for Applications in Li-Ion Batteries and Supercapacitors. Membranes.

[15] Huang J., Jiang H., Wu F., Xiong X.J., Han K. (2022). Natural Halloysite Nanotubes Coated Commercial Paper or Waste Newspaper as Highly-Thermal-Stable Separator for Lithium-Ion Batteries. Adv. Mater. Technol..

[16] Yang C., Xiao Y., You H., Liu Z., Liu Q., Zang L. (2023). A facile fabrication of lightweight current collector based on used newspaper for flexible zinc-ion hybrid supercapacitors. J. Alloys Compd..

[17] Nahar A., Akbor M.A., Rahman M.A., Ferdous Z., Hasan M.R., Kamruzzaman S., Shristy N.T., Saha P., Akthar U.S., Bashar M.S. (2025). Enhanced electrochemical performance of waste newspaper derived activated carbon aerogel electrode for the supercapacitor. Results Eng..

[18] Lee M.J., Han C.M., Hong H.W., Jung J.H., Lee K.S. (2025). Novel methods and approaches for supercapacitor separator using waste newspaper. J. Chin. Chem. Soc..

[19] Coelho B.J., Pinto J.V., Martins J., Rovisco A., Barquinha P., Fortunato E., Baptista P.V., Martins R., Igreja R. (2023). Parylene C as a Multipurpose Material for Electronics and Microfluidics. Polymers.

[20] Lee Y.S., Yoon J.H., Raji A., Baek S.Y., Choi Y.S., Lee J.H., Gasonoo A., Lee J.H. (2022). Optical and Electrical Characterization of Visible Parylene Films. Materials.

[21] Fang W., Ma Z., Lv X., Liu J., Pei W., Geng Z. (2022). Flexible terahertz metamaterial biosensor for label-free sensing of serum tumor marker modified on a non-metal area. Opt. Express.

[22] Zhang H., Lv X., Huang B., Cheng C., Zhang Z., Zhang Z., Fang W., Zhang H., Chen R., Huang Y. (2022). In Situ Regeneration of Silicon Microring Biosensors Coated with Parylene C. Langmuir.

[23] Lima R.M.A.P., Reis G.S., Lassi U., Lima E.C., Dotto G.L., Oliveira H.P. (2023). Sustainable Supercapacitors Based on Polypyrrole-Doped Activated Biochar from Wood Waste Electrodes. C.

[24] Liu G., Huang Z., Xu J., Lin T., Zhang B., He P. (2024). MnO_2_ Nanoparticles Decorated PEDOT:PSS for High Performance Stretchable and Transparent Supercapacitors. Nanomaterials.

[25] Alizadeh A., Ahmadi E. (2025). Polyaniline and MOF5 functionalized mesoporous silica composite as electrode for high performance symmetric supercapacitor. Sci. Rep..

[26] Tadesse M.G., Ahmmed A.S., Lübben J.F. (2024). Review on Conductive Polymer Composites for Supercapacitor Applications. J. Compos. Sci..

[27] Butt A.S., Qaiser A.A., Abid N., Mahmood U. (2022). Novel polyaniline–polyethersulfone nanofiltration membranes: Effect of in situ polymerization time on structure and desalination performance. RSC Adv..

[28] Mücke B.E., Rossignatti B.C., Abegão L.M.G., Barbosa M.S., Mello H.J.N.P. (2024). Optimized Drop-Casted Polyaniline Thin Films for High-Sensitivity Electrochemical and Optical pH Sensors. Polymers.

[29] Badry R.B., Elhaes H., Ibrahim A., Refaat A., Ibrahim M.A. (2024). Investigating the electronic properties and reactivity of polyaniline emeraldine base functionalized with metal oxides. Sci. Rep..

